# Investigating the effects of transcranial alternating current stimulation on primary somatosensory cortex

**DOI:** 10.1038/s41598-020-74072-2

**Published:** 2020-10-13

**Authors:** Nicoletta Manzo, Andrea Guerra, Margherita Giangrosso, Daniele Belvisi, Giorgio Leodori, Alfredo Berardelli, Antonella Conte

**Affiliations:** 1 IRCCS NEUROMED, Via Atinense, 18, 86077 Pozzilli, IS Italy; 2 Department of Human Neuroscience, Sapienza University of Rome, Viale dell’Università 30, 00185 Rome, Italy

**Keywords:** Neuroscience, Physiology, Neurology

## Abstract

Near-threshold tactile stimuli perception and somatosensory temporal discrimination threshold (STDT) are encoded in the primary somatosensory cortex (S1) and largely depend on alpha and beta S1 rhythm. Transcranial alternating current stimulation (tACS) is a non-invasive neurophysiological technique that allows cortical rhythm modulation. We investigated the effects of tACS delivered over S1 at alpha, beta, and gamma frequencies on near-threshold tactile stimuli perception and STDT, as well as phase-dependent tACS effects on near-threshold tactile stimuli perception in healthy subjects. In separate sessions, we tested the effects of different tACS montages, and tACS at the individualised S1 μ-alpha frequency peak, on STDT and near-threshold tactile stimuli perception. We found that tACS applied over S1 at alpha, beta, and gamma frequencies did not modify STDT or near-threshold tactile stimuli perception. Moreover, we did not detect effects of tACS phase or montage. Finally, tACS did not modify near-threshold tactile stimuli perception and STDT even when delivered at the individualised μ-alpha frequency peak. Our study showed that tACS does not alter near-threshold tactile stimuli or STDT, possibly due to the inability of tACS to activate deep S1 layers. Future investigations may clarify tACS effects over S1 in patients with focal dystonia, whose pathophysiology implicates increased STDT.

## Introduction

The ability to perceive a single tactile stimulus can be measured with near-threshold tactile stimuli detection^1–3^, while the temporal discrimination can be measured with the somatosensory discrimination threshold (STDT) technique^4,5^. Previous neurophysiological and neuroimaging studies have shown that the abilities to perceive single tactile stimuli and to temporally discriminate paired electrical tactile stimuli are mainly encoded in primary somatosensory cortex (S1)^2,4–10^ and are influenced by alpha and beta cortical rhythms^3,11–16^.

Cortical rhythms can be modulated with transcranial alternating current stimulation (tACS), which is a non-invasive brain stimulation (NIBS) technique that allows neuronal oscillations to be entrained by inducing coherent changes in the firing and timing of neuron populations^17–20^. While a previous study found that tACS modulates near-threshold tactile stimuli perception^21^, another study showed no effect^22^. Only one study has investigated the effect of tACS on STDT, and this study found that tACS delivered at alpha frequency did not affect STDT^23^. Therefore, the effects of tACS applied over S1 on near-threshold tactile stimuli perception and STDT are still unclear. Studies on the interference exerted by tACS on ongoing S1 activity may clarify the mechanisms underlying tACS applied over S1 in healthy subjects.

These studies are important for possible therapeutic neuromodulation protocols in neurological conditions whose pathophysiology implicates abnormal sensory processing in S1 (i.e. focal dystonia), as demonstrated by the increased STDT^24^.

The aim of this study was to investigate the effects of tACS delivered over S1 on STDT and near-threshold tactile stimuli perception. For this purpose we tested the effects of tACS delivered at various frequencies (alpha, beta, and high gamma) on STDT^11,12^ and near-threshold tactile stimuli perception^3,13^. Possible phase-dependent effects of tACS on near-threshold tactile stimuli were also investigated. In a separate experiment we targeted S1 according to a modelling-based montage and tested the effects of tACS on the same parameters. Finally, since tACS effects are maximal when stimulation frequency matches the endogenous rhythm^17,19^, we recorded the individual EEG activity of S1 and investigated the effects of tACS delivered at the individualised μ-alpha frequency peak.

## Results

### Experiment 1: Effects of tACS on STDT and near-threshold tactile stimuli perception

**Figure 1:**
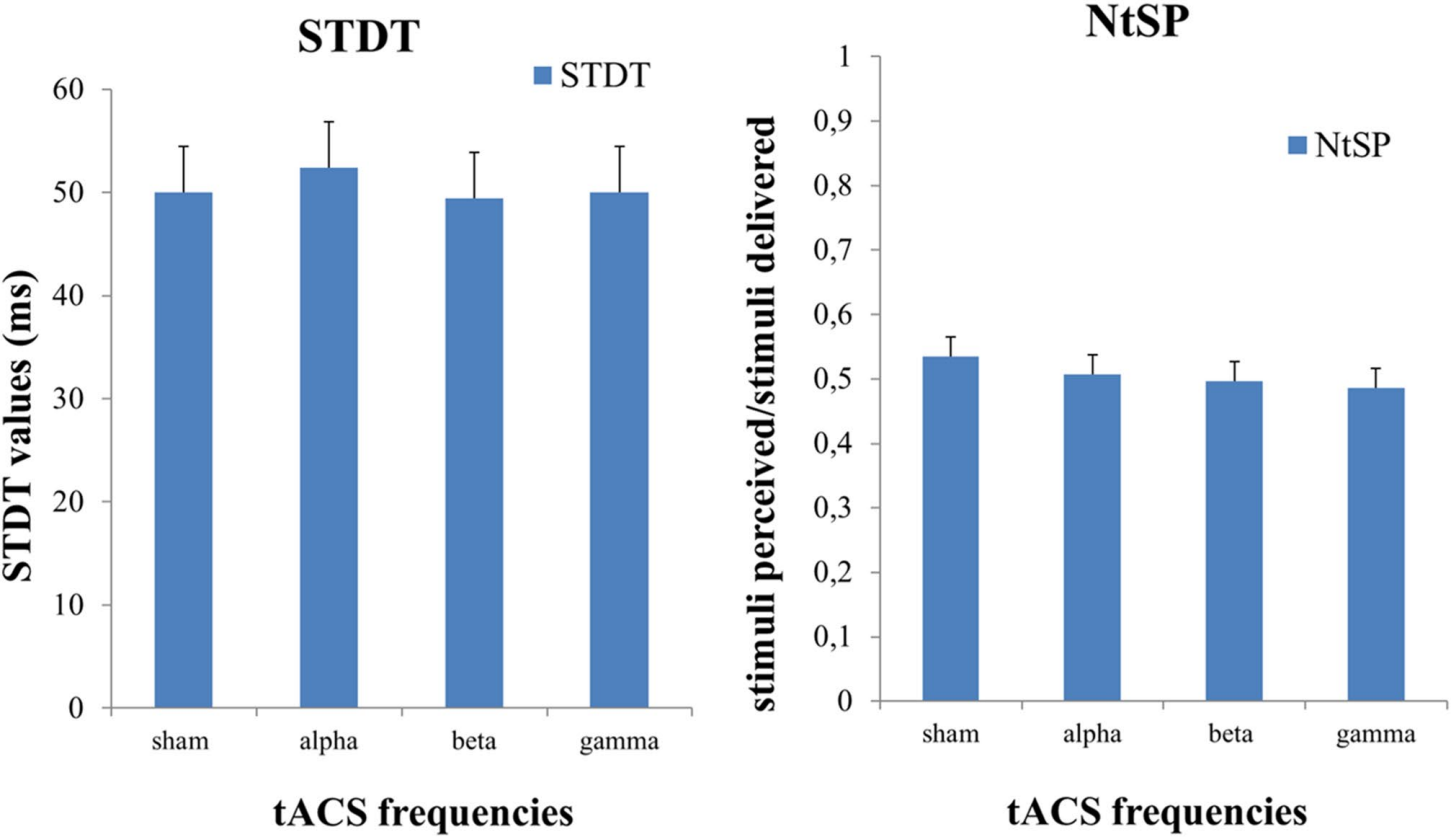
Effects of tACS frequency on somatosensory temporal discrimination (STDT) and near- threshold tactile stimuli perception (NtSP). Alpha, beta, and gamma tACS did not induce significant changes on STDT (left panel) or NtSP (right panel). Sham tACS was used as a control.

**Figure 2:**
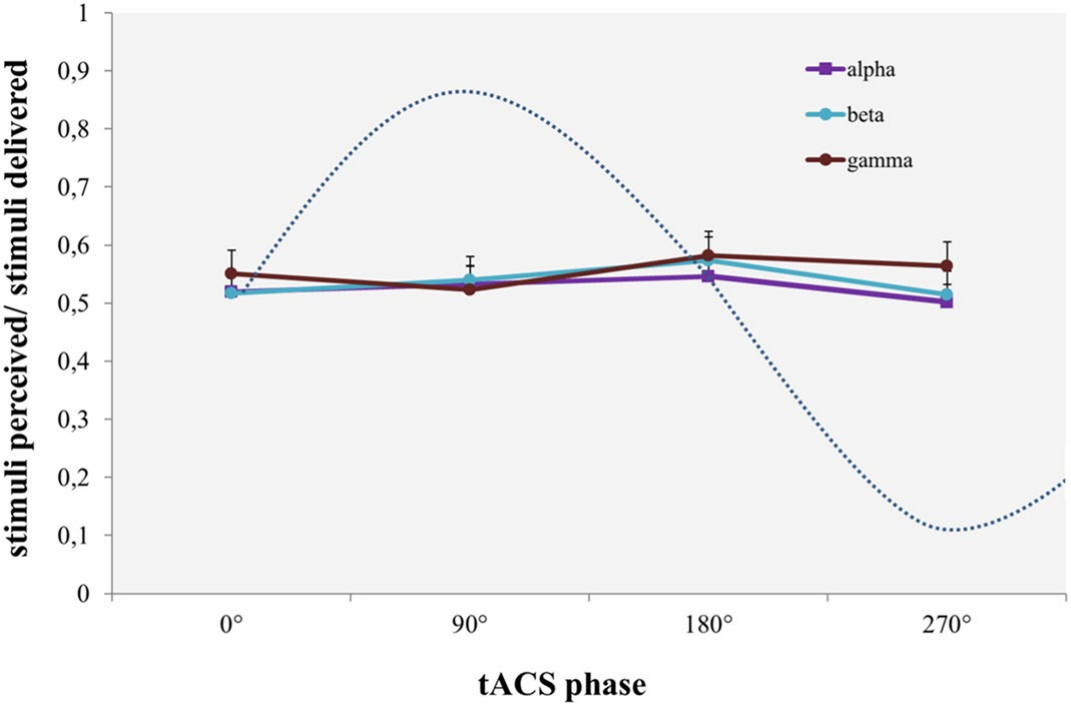
Effects of tACS phase on NtSP. tACS did not modify near-threshold stimuli perception (NtSP) in a phase-dependant manner. The violet curve represents alpha tACS, the blue curve represents beta tACS and the red curve represents gamma tACS. The dashed line represents an exemplary tACS sinusoid curve. Error bars represent standard error.

Sensory thresholds and STDT values (Fig. 1) were comparable between alpha (sensory threshold: 1.97 ± 0.42; STDT: 46.24 ± 18.94), beta (sensory threshold: 2.01 ± 0.44; STDT: 48.42 ± 21.05), gamma (sensory threshold: 2.02 ± 0.48; STDT: 45.47 ± 14.81), and sham tACS (sensory threshold: 1.95 ± 0.46; STDT: 45.28 ± 15.13), as shown by the non-significant factor “FREQUENCY” in rmANOVAs (sensory thresholds: F(3,48) = 1.229, *p* = 0.31; STDT: F(3,48) = 0.319, *p* = 0.73). The non-significant effect of the factor “FREQUENCY” was confirmed by the analysis performed on STDT values normalised to sham (F(2,32) = 0.231, *p* = 0.79). Additionally, tACS did not modify near-threshold stimuli perception, either phase-independently (Fig. 1) or in a phase-dependant manner (Fig. 2), as indicated by the non-significant factors “FREQUENCY” (F(3,48) = 1.147, *p* = 0.34) and “PHASE” (F(3,48) = 2.935, *p* = 0.07), and the lack of a “FREQUENCY” × “PHASE” interaction in rmANOVA (F(6,96) = 0.601, *p* = 0.72).

Analysis of sham-normalised near-threshold stimuli perception values also confirmed no effect of the factor “FREQUENCY” (F(2,32) = 0.24, *p* = 0.78). The analysis performed to evaluate whether near-threshold stimuli perception changed over time within each block showed a non-significant effect of the factor “TIME” (F(1,16) = 0.024, *p* = 0.87) and the lack of a “TIME” × “FREQUENCY” interaction (F(3,48) = 0.361, *p* = 0.78).

### Experiment 2: Effects of tACS using a modelling-based montage

Sensory thresholds and STDT values were comparable between experimental sessions 1 and 2, as suggested by the non-significant factor “MONTAGE” (sensory thresholds: F(1,16) = 1.87347, *p* = 0.19; STDT: F(1,16) = 0.433, *p* = 0.52) and the lack of a “MONTAGE” × “FREQUENCY” interaction (sensory thresholds: F(3,48) = 0.15, *p* = 0.89; STDT: F(3,48) = 0.159, *p* = 0.8) in rmANOVAs. Additionally, neither sensory thresholds nor STDT changed during alpha (sensory threshold: 1.77 ± 0.5; STDT: 50 ± 17.8), beta (sensory threshold: 1.77 ± 0.51; STDT: 52.35 ± 21.8), gamma (sensory threshold: 1.74 ± 0.5; STDT: 49.41 ± 17.31), or sham tACS (sensory threshold: 1.74 ± 0.45; STDT: 52.6 ± 16.5), as shown by the non-significant factor “FREQUENCY” (sensory thresholds: F(3,48) = 0.295, *p* = 0.68; STDT: F(3,48) = 0.344, *p* = 0.65). The non-significant effect of the factor “FREQUENCY” was further confirmed by the analysis performed on STDT values normalised to sham (F(2,32) = 0.237, *p* = 0.7). Similarly, near-threshold tactile stimuli perception was not influenced by the montage used (“MONTAGE” × “FREQUENCY”: F(2,32) = 3.082, *p* = 0.09; “MONTAGE” × “PHASE”: F(3,48) = 0.537, *p* = 0.65; “MONTAGE” × “FREQUENCY” × “PHASE”: F(6,96) = 0.2423, *p* = 0.96). Accordingly, tACS did not modulate near-threshold tactile stimuli perception in a frequency- or phase-dependant manner even in this experiment (for the factor “FREQUENCY”: F(3,48) = 1.04, *p* = 0.35; for the factor “PHASE”: F(3,48) = 0.59, *p* = 0.62; and for the “FREQUENCY” × “PHASE” interaction: F(6,96) = 1.167, *p* = 0.3317). Analysis of sham-normalised near-threshold stimuli perception values also confirmed no effect of the factor “FREQUENCY” (F(2,32) = 0.19, *p* = 0.74). The analysis performed to evaluate whether near-threshold stimuli perception changed over time within each block showed a non-significant factor “TIME” (F(1,16) = 0.962, *p* = 0.34) and the lack of a “TIME” × “FREQUENCY” interaction (F(3,48) = 0.196, *p* = 0.9).

### Experiment 3: Effects of tACS at the individualised μ-alpha rhythm frequency peak

Sensory threshold and STDT were comparable between individualised (sensory threshold: 1.665 ± 0.6; STDT: 53.5 ± 19) and fixed μ-alpha stimulation (sensory threshold: 1.77 ± 0.5; STDT: 52.944.4 ± 18.9), as shown by paired t-tests. The effects of tACS on near-threshold tactile stimuli perception did not differ when tACS stimulation was performed at a fixed frequency of 12 Hz (experiment 1) or at the individualised μ-alpha frequency, as shown by the non-significant factors “FREQUENCY” (F(1,16) = 1.552, *p* = 0.23) and “PHASE” (F(3,48) = 2.106, *p* = 0.11), and the lack of a “FREQUENCY” × “PHASE” interaction (F(3,48) = 0.288, *p* = 0.83) in rmANOVA. Moreover, tACS delivered at the individualised μ-alpha frequency peak did not determine phase-dependent effects on near-threshold tactile stimuli perception (for the factor “PHASE”: F(3,48) = 1.146, *p* = 0.3497).

## Discussion

In the present study we demonstrated that tACS failed to modify STDT and near-threshold tactile stimuli perception, not only when delivered at alpha frequency^22,23^, but even at beta and gamma frequencies. Moreover, our study showed that the phase of tACS stimulation over S1 did not affect near-threshold tactile stimuli perception. In addition, we demonstrated that tACS delivered using a modelling-based montage did not modify STDT or near-threshold tactile stimuli perception. Finally, tACS did not modify near-threshold tactile stimuli detection and STDT, even when delivered at the individualised μ-alpha frequency peak.

Our first result demonstrating that tACS does not modulate STDT regardless of stimulation frequency is in line with previous evidence showing no effect of 10 Hz tACS applied over S1 on STDT^23^. Our finding that tACS did not affect overall near-threshold tactile stimuli perception contrasts with that of Sliva et al.^21^, who showed a decreased performance in near-threshold stimuli perception during alpha tACS. However, this effect was present only with baseline-corrected detection rates, but not for absolute detection rates, as was used in our study and in that by Gundlach et al.^22^.

To avoid possible confounding as an explanation for the lack of effect of tACS stimulation on both STDT and near-threshold tactile stimuli perception, our procedure adopted several precautions. First, in order to reduce task interference and ensure a proper blinding of participants, we lowered tACS intensities to avoid uncomfortable skin or visual sensations. In addition, sham tACS was adopted as a control condition. We randomized the four tACS frequencies and stimulation phases for each subject in order to avoid a learning effect on STDT or near-threshold tactile stimuli perception. To ensure that subjects maintained an appropriate attention level throughout the task, we included two “no tactile stimulus” trials after every 10 stimuli in our procedure investigating near-threshold tactile stimuli perception. STDT bias attributable to a lack of attention was excluded by using “catch trials”^4,25^.

One possible methodological reason which may explain the lack of tACS effect on near-threshold tactile stimuli perception and STDT relates to the suboptimal targeting of S1. However, we believe this is unlikely for several reasons. First, we adopted the same montage used in previous studies showing significant effects of tACS over S1^21,22^. In addition, although we did not specifically localise S1 with TMS^26^ or magnetic resonance imaging techniques for each participant, the size of tACS electrodes (5 × 5 cm; area of 25 cm^2^) was large enough to ensure that the current reached S1. Furthermore, tACS stimulating electrodes were applied to the same region from which we recorded μ-alpha during task execution, a rhythm which is known to correlate with STDT performance and tactile stimuli perception^3,12^, as shown by previous studies with magnetoencephalography (MEG) and EEG^12^.

A further possible explanation for the lack of tACS effect on STDT and near-threshold tactile stimuli perception is that the frequency used did not correspond with the oscillation within S1. To verify this issue, we adjusted the stimulation protocol to the individualised μ-alpha frequency peak in experiment 3. However, we again found no effect on near-threshold tactile stimuli perception. Thus, since stimulation was performed at a functionally relevant endogenous frequency, our results exclude that tACS over S1 failed to modulate STDT and near-threshold stimuli perception due to the targeted neuronal population specificity.

To explain the lack of tACS effect on STDT and near-threshold tactile perception we considered the possibility that the stimulation intensity used was too low to affect behavioural tasks. Previous studies in humans using current intensities of 0.5–0.7 mA have demonstrated brain oscillation entrainment in occipital areas and the primary motor cortex (M1)^27–31^. In addition, when tACS matches endogenous frequency, as in experiment 3, entrainment occurs even at very low stimulation intensities^18,32^. Recent studies have found that scalp-applied currents-compared to the conventional tACS approach-should exceed 4–6 mA to achieve measurable effects on transmembrane potential spikes^33–35^. Although we used the highest tolerable intensity in our study, we cannot exclude the possibility that higher current intensities of alpha and beta tACS S1 stimulation may be effective in modulating STDT and near-threshold tactile perception. Nevertheless, since the mean intensity of gamma tACS was 1 mA, we may exclude that the lack of gamma tACS effect on STDT and near- threshold tactile perception was due to stimulation intensity.

Alternatively, we may theorise that the lack of S1 tACS effects on STDT and near-threshold stimuli perception depends on S1 architecture. Evidence in animals has shown that the electrical activity evoked by a sensory stimulus simultaneously involves specific layers of sensory cortices, namely L4, L5, and L6^36,37^. Similarly, STDT underlies interneuronal inhibitory circuits in L5 and L6 layers in S1^37,38^. To date, the specific features and location of neurons entrained by tACS are not fully understood, but recent data has suggested that candidate elements are interneurons in L2 or L3, or cells located in L1 such as single-bouquet and elongated neurogliaform cells, which are reciprocally connected with L2 and L3 interneurons^39,40^. Thus, we may speculate that tACS is not able to reach deeper layers within S1 (i.e. L4, L5, and L6) where neurons that are functionally involved in STDT and near-threshold tactile stimuli encoding lie. Hence, we suggest that intrinsic features of tACS, together with the peculiar architecture of S1^36^ may explain why tACS failed to modulate STDT and near-threshold tactile stimuli perception. It is also possible that tACS entrains oscillatory neurons in S1, but the amount of this effect was not sufficient to determine significant modifications in the somatosensory behavioural task.

One remaining question is why an ample body of studies using tACS applied over the primary motor cortex (M1) showed an effect of tACS on motor neurophysiological parameters encoded in M1 in healthy subjects^28–30,40–45^. It is important to consider that the effectiveness of an externally applied field may be influenced by a variety of factors, including neuronal density and architecture, alignment of dendrites and axons relative to the induced field, type and distribution of ion channels in the neurons, degree of myelination, and glia density. These factors, which change in different brain regions, probably differ between S1 and M1^37,46–48^ thus explaining, at least in part, the different effectiveness of tACS on these two cortical areas.

In conclusion, we have demonstrated that tACS applied over S1 does not alter behavioural tasks that rely on neural processing in S1, such as STDT, or near-threshold tactile stimuli perception. These findings may suggest that S1 tACS only entrains superficial cortical layer neurons that do not play a major role in STDT or near-threshold tactile stimuli encoding. However, it cannot be ruled out that higher tACS intensities, smaller electrodes or multifocal modalities of stimulation may allow to modulate these tasks. Finally, although our study showed that in healthy subjects tACS applied over S1 fails to modify sensory performance, future studies are needed to investigate possible tACS effects on perception in neurological conditions implying abnormal sensory processing in S1, such as focal dystonia. It is possible that in dystonia, which is characterised by an increased STDT, tACS delivered over S1 at specific frequencies may normalize or improve STDT processing.

## Methods

### Participants

Seventeen healthy volunteers (8 female, mean age ± SD: 28.5 ± 2.6) were enrolled in the study. Participants had no history of neuropsychiatric disorders, were not taking drugs acting on the central nervous system at the time of the experiments, and had no contraindications to NIBS according to the latest international guidelines^49^. All participants except two were right-handed, as evaluated by the Edinburgh Handedness Inventory. All experimental procedures were performed in accordance with the Declaration of Helsinki. The study was approved by the ethics committee of Sapienza, University of Rome, and informed consent was signed by all study participants.

### Sensory threshold

We first determined each participant’s individual sensory threshold, defined as the intensity at which the participant reported feeling 50% of tactile electrical stimuli delivered according to the staircase method^50^. Electric suprathreshold stimuli were applied to the index finger of the dominant hand through surface electrodes, with the anode located 0.5 cm distally from the cathode, using a constant current stimulator (Digitimer DS7AH) while participants looked at a central point in front of them. Participants were required to verbally report whether they had perceived a pulse. The rater manually set the intensity, and the threshold for the main experiment was defined as 120% of the sensory threshold.

### Somatosensory temporal discrimination threshold (STDT)

STDT was investigated by delivering paired stimuli starting with an interstimulus interval (ISI) of 0 ms (simultaneous pair) and progressively increasing the ISI by 10 ms using a previously described experimental procedure^4,25^. Paired electrical stimuli consisted of square-wave electrical pulses delivered with a constant current stimulator (Digitimer DS7A) through surface electrodes placed with the anode located 0.5 cm distally to the cathode. The surface electrodes were applied on the distal phalanx of the index finger of the dominant hand. Stimulation intensity was defined for each subject by delivering a series of stimuli at an increasing intensity, starting at 2 mA and increasing in increments of 0.5 mA. The intensity used for STDT was the minimum intensity perceived in 10 out of 10 consecutive stimuli^4,25^. Before STDT testing started, subjects familiarised themselves with the task to achieve a stable performance. Subjects were asked to verbally report whether they had perceived a single stimulus or two temporally separate stimuli. The first of three consecutive ISIs at which participants recognised the stimuli as temporally separate was considered the STDT. To maintain a constant attention level during the test and to minimise the risk of perseverative responses, the experimental procedure included “catch” trials consisting of a single stimulus delivered randomly. Each session included four separate blocks. STDT was defined as the average of the four STDT values and was entered into data analysis.

### Perception of near-threshold tactile stimuli: task and analysis

The ability to detect near-threshold tactile stimuli was investigated by delivering a single stimulus on the index finger of the dominant hand at an intensity of 120% of the sensory threshold, and subjects were asked to report whether they had perceived the stimulus. Tactile stimuli consisted of square-wave electrical pulses delivered with a constant current stimulator (Digitimer DS7A) through surface electrodes placed with the anode located 0.5 cm distally to the cathode. The surface electrodes were applied on the distal phalanx of the index finger. Before the perception task started, subjects underwent a training block to familiarise themselves with the task. Each block consisted of 120 tactile single stimuli, separated by an ISI of 10 s. Perception task performance was measured as the ratio between the number of stimuli perceived/the number of stimuli delivered during each tACS frequency stimulation. A rater manually recorded participant responses and analysis was performed offline.

### Transcranial alternating current stimulation (tACS)

**Figure 3:**
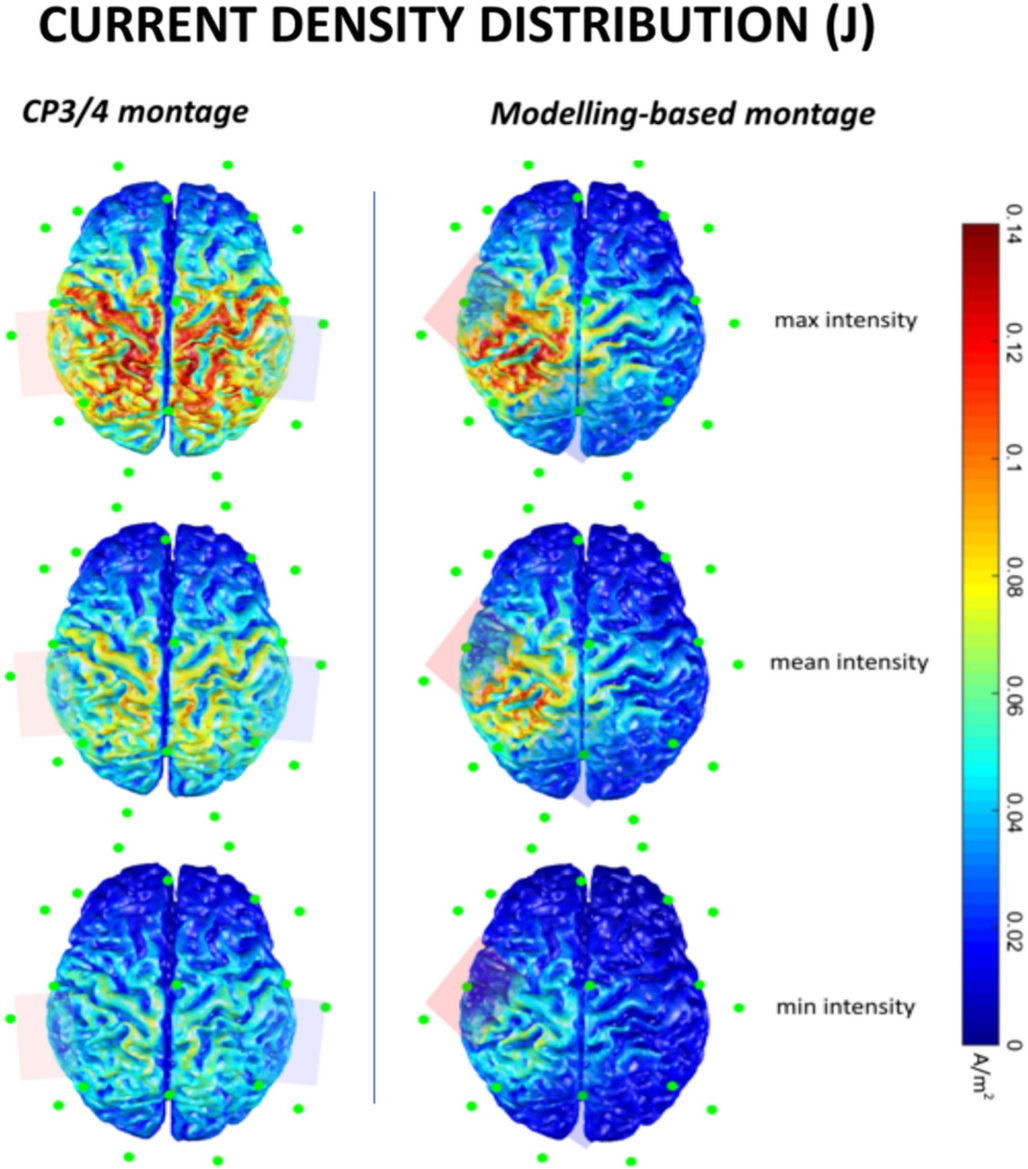
Estimated current density distribution produced by stimulating with various montages at the minimum, maximum and mean intensities used, according to the MATLAB toolbox Comets2 (https://www.cometstool.com). In experiment 1 tACS electrodes were placed over Cp3/Cp4 (left panel). In experiment 2 one electrode was placed over CP3 and the other was positioned over Pz, and both electrodes were specifically oriented (right panel).

Transcranial alternating current stimulation (tACS) was delivered through conductive rubber electrodes (5 × 5 cm in size) enclosed in saline-soaked sponges using BrainSTIM (EMS, Italy). In order to ensure targeted S1 stimulation, two different tACS montages were used. Consistent with previous S1 tACS studies^22,51^, in the main experimental session (see below) the electrodes were placed over CP3 and CP4, according to the 10–20 international system (CP3/4 montage, Fig. 3). Notably, the electrode connected to the positive pole of the stimulator was always contralateral to the dominant hand, so that in left-handed subjects it was placed over CP4. A different montage was used in experiment 2 and 3 (see below). Using the MATLAB toolbox Comets2 (https://www.cometstool.com)^52^, we estimated the current density distribution produced by stimulating with various montages at 1 mA intensity. We then identified the montage that most precisely targeted S1. As a result, the electrode connected to the positive pole of the stimulator was placed over CP3, while the other was positioned over Pz, and both electrodes were specifically oriented (modelling-based montage, Fig. 3). Set-up was optimized by keeping the impedance below 10 kΩ as measured by the stimulation device. Electrodes were secured in place using rubber strips around the head. We delivered tACS at four different frequencies: alpha (12 Hz), beta (20 Hz), gamma (70 Hz), and at the individualised μ-alpha frequency. Ramp-up and ramp-down periods of tACS lasted 3 s in all conditions. A sham tACS was used as a control, which consisted of ramp-up and down periods and 1 s of stimulation at 70 Hz. The intensity of tACS was first set at 1 mA (peak-to-peak). However, if participants experienced unpleasant sensations, such as visual or skin discomfort, the stimulation intensity was gradually lowered in steps of 0.05 mA until the discomfort disappeared. This procedure allowed us to exclude placebo or attentional effects due to the perception of stimulation and ensured that patients had the same sensation in each stimulation condition. The stimulation intensities used were 0.48 ± 0.09 mA for alpha, 0.57 ± 0.13 mA for beta, and 0.45 ± 0.12 mA for the individualised μ-alpha tACS. Gamma tACS intensity did not need to be adjusted in any participant. Notably, these intensities were the highest possible that avoided the experience of any uncomfortable sensations, which would have interfered with task execution.

### Transcranial alternating current stimulation phase recording and analysis

To investigate possible effects of tACS phase on near-threshold stimuli perception, tactile stimuli were delivered at four different phases of the tACS waveform during alpha, beta, gamma, and individualised μ-alpha stimulation. Following the methods described in Guerra et al., 2016, the tACS signal was streamed to a battery-operated interface connected to the current stimulator (EMS, Italy), recorded in real time using Signal software (Cambridge Electronic Design). The instantaneous phase of the tACS waveform was calculated using an in-house written script. Four specific tACS phases were targeted (0 degrees (°), 90°, 180°, and 270°)^42^. Since cortical processing of peripheral electrical stimuli starts 20 ms after stimulus onset, stimuli were delivered 20 ms before the targeted phase (30 stimuli for each phase) in a randomized order using a custom-made script in Signal. Moreover, since the duration of one tACS cycle differed based on the stimulation frequency, we delivered electrical stimuli according to the four tACS phases tested, taking into account the duration of each cycle. The rater manually recorded stimuli perceived by the subjects online. Data were analysed offline in order to measure the number of stimuli perceived in each phase of tACS stimulation.

### EEG recording: determination of the individualised μ-alpha rhythm frequency peak

We identified the individual μ-alpha rhythm frequency peak of each participant by measuring somatosensory event-related desynchronization (ERD) using a method similar to that described by Gundlach et al. (2017)^51^. EEG was sampled at 5 kHz (NeurOne, Bittium, Finland) from channel CP3 or CP4 (10–20 layout), which were online referenced to POz channels, in right- and left-handed participants respectively. The Fpz channel was used as the ground. Impedance was kept below 5 kΩ. Subjects were fully relaxed with their eyes open. During continuous EEG recording we delivered 120 electric single stimuli with an ISI of 10 s using the same methodological procedure as previous experimental sessions. EEG was pre-processed and analysed immediately after the recording using custom script with EEGLAB^53^ and Fieldtrip^54^ toolboxes in Matlab (R2017b, The Mathworks, USA). Continuous EEG down-sampled to 1 kHz, detrended, notch filtered (48–52 Hz), bandpass filtered from 1–95 Hz, and segmented into 150 3-s epochs (− 1500 to 1500 ms) around each electric stimulus. EEG epochs were then used to calculate the time–frequency power spectrum between 5 and 35 Hz using time-domain complex Morlet wavelet convolution with 0.1 Hz resolution and using five cycle long wavelets. The time–frequency power spectra for each epoch were decibel-converted with respect to the average value of the baseline period (− 600 to − 300 ms) and averaged across epochs. For each participant, we defined the individual μ-alpha rhythm frequency peak as the frequency with the maximum ERD (i.e. the maximum decrease in power) in the 8–14 Hz frequency band between 200 and 600 ms poststimulus. The average μ-alpha rhythm frequency peak was 11 ± 1.95 Hz (mean ± SD).

### Experimental paradigm

All 17 participants underwent three experimental sessions that took place at least one week apart in order to exclude any possible carry-over effects. In each session, tACS was applied at different frequencies in separate and randomised blocks. The tACS inter-block interval was twice as long as the duration of the previous stimulation in order to avoid habituation with repeated stimulation and to exclude after effects. In the first experimental session (experiment 1: effects of tACS on STDT and near-threshold tactile stimuli perception), we studied the effects of tACS on STDT and near-threshold tactile stimuli perception during alpha, beta, gamma, and sham tACS delivered over S1 contralateral to the dominant hand by using the CP3/4 montage. During alpha, beta, and gamma tACS, the effect of tACS phase on near-threshold tactile stimuli perception was also investigated.

In the second experimental session (experiment 2: effects of tACS using a modelling-based montage), we investigated the effects of tACS on STDT and near-threshold tactile stimuli perception using a modelling-based montage.

**Figure 4:**
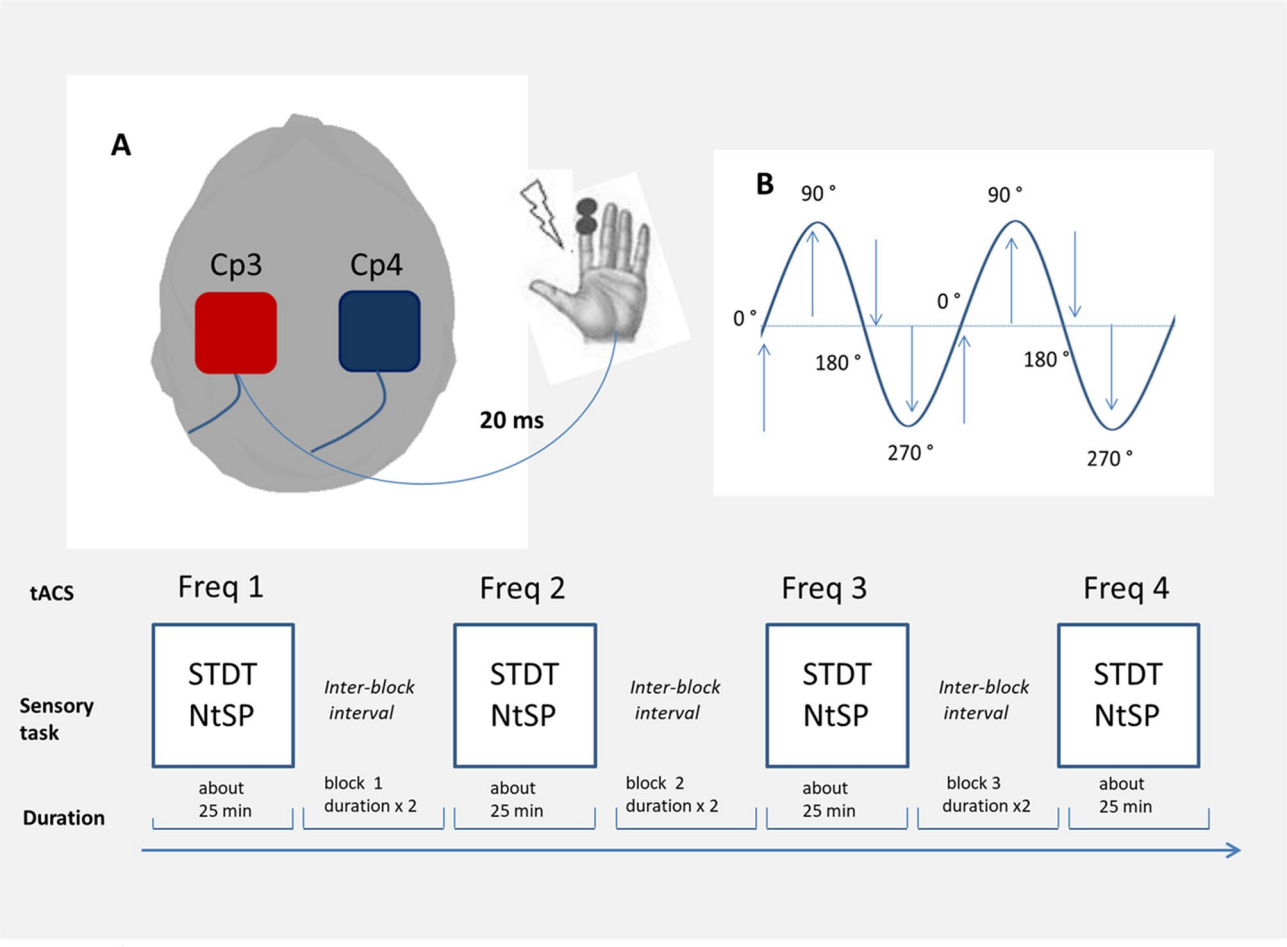
Experimental set up. (**A**) Tactile stimuli for near-threshold tactile stimuli perception (NtSP)and somatosensory temporal discrimination threshold (STDT) were delivered on the index finger of the dominant hand during tACS. tACS was delivered over CP3/4 at four different frequencies (alpha, beta, gamma and sham) in a randomized order. Each block lasted about 25 min and the inter-block interval was twice as long as the previous block duration (except after sham). (**B**) During alpha, beta, and gamma tACS, tactile stimuli were delivered at four different phases of tACS stimulation (0°, 90°, 180°, and 270°) to investigate the effect of tACS phase on NtSP.

In the third experimental session (experiment 3: effects of tACS at the individualised μ-alpha rhythm frequency peak), we recorded cerebral activity with EEG to identify the individual μ-alpha rhythm frequency peak of oscillatory activity within S1 using the same montage as experiment 2. Subsequently, STDT and tactile stimuli perception were investigated during tACS applied over S1 at the individualised μ-alpha frequency. In addition, in experiment 3 tACS phase was recorded and phase-dependent effects were tested (Fig. 4).

### Statistical analysis

G* Power software was used for power analysis. Based on previous results on tactile perception modulation^4,55^ with a sample of 16 subjects and an alpha error probability of 0.05, study power was 0.9.

Repeated measures analysis of variance (rmANOVA) with “FREQUENCY” as a factor (levels: alpha, beta, gamma, and sham) was used to investigate possible effects of tACS on sensory threshold and STDT. A further rmANOVA with “FREQUENCY” as a factor (levels: alpha, beta and gamma) was performed on STDT and near-threshold stimuli perception values normalised to those obtained in the sham session. To evaluate whether possible effects of tACS changed over time during each block, we also performed a rmANOVA with “TIME” as a factor considering the first and second half of each block separately (levels: T1, T2). To evaluate the effects of tACS on near-threshold stimuli perception we performed rmANOVA with “PHASE” (levels: 0°, 90°, 180°, and 270°) and “FREQUENCY” (levels: alpha, beta, and gamma) as factors. To verify whether tACS effects depended on different electrode montages, we also compared the effects produced by tACS stimulation using CP3/4 to those obtained using the modelling-based montage. We first performed rmANOVA with “MONTAGE” (levels: CP3/4, modelling-based) and “FREQUENCY” (levels: alpha, beta, gamma, and sham) as factors to contrast sensory thresholds and STDT between experiment 1 and 2. We then performed rmANOVA with “MONTAGE”, “FREQUENCY” (levels: alpha, beta, and gamma), and “PHASE” as factors of near-threshold stimuli perception. To compare the effects of tACS delivered at the individualised μ-alpha frequency to those obtained at the fixed alpha frequency of 12 Hz, we used rmANOVA with “FREQUENCY” (levels: individualised μ-alpha and fixed alpha) and “PHASE” as factors. We also specifically tested whether tACS delivered at the individualised μ-alpha frequency modulated near-threshold tactile stimuli detection by using rmANOVA with the factor “PHASE” on data recorded in experiment 3. The level of significance was set at *p* < 0.05 for all analyses. Data were analysed using SPSS Statistics for Windows (version 25.0; IBM).
